# Supplementary material for: MetaRibo-Seq measures translation in microbiomes
Source: Nat Commun. 2020 Jun 29;11:3268. doi: 10.1038/s41467-020-17081-z (PMC7324362; doi:10.1038/s41467-020-17081-z)
Supplement: Supplementary file 10 — Supplementary Data 7 [file 41467_2020_17081_MOESM10_ESM.zip › File2/Confidence_High_Taxonomy/67996_out.krona.html]

Javascript must be enabled to view this page.

members
magnitude
magnitudeUnassigned
count
unassigned
taxon
rank

67996\_out

7

superkingdom
7
2

phylum
7
1239

7
186801
class

order
7
186802

6
186803
family

genus
2
33042


SRS054905\_contig\_number\_contig-100\_197.146649SRS148424\_contig\_number\_contig-100\_2066.187688
species
410072
2

1
841
genus

species

SRS043667\_contig\_number\_contig-100\_633.46198
1
166486

genus
2
28050

1946726
2

SRS075078\_contig\_number\_27505SRS077231\_contig\_number\_contig-100\_19442.100922
species

1
572511
genus


SRS053398\_contig\_number\_6694
species
1
40520

31979
1
family

genus
1485
1

1
59620
species

SRS075773\_contig\_number\_17401
